# Supplementary material for: Jumbo circular extrachromosomal elements of methane-oxidizing archaea with variably extensive metabolic and defense gene repertoires
Source: Nat Commun. 2026 Jun 23;17:7878. doi: 10.1038/s41467-026-74423-z (PMC13444090; doi:10.1038/s41467-026-74423-z)
Supplement: Supplementary file 2 [file 41467_2026_74423_MOESM2_ESM.pdf]

Description of Additional Supplementary Files:

Supplementary Data 1: Stats of 14 new complete ECE genomes.

Supplementary Data 2: Stats of 11 circular complete *Methanoperedens* genomes.

Supplementary Data 3: Co-occurrence of ECEs and predicted *Methanoperedens* hosts in more than one sample.

Supplementary Data 4: Sequence- and structure-based annotations of ECE proteins.

Supplementary Data 5: Gene expression of 283kb_39_10 and 233kb_41_10 in a wetland sediment column.

Supplementary Data 6: Gene presence and absence across the ECEs and *Methanoperedens*.
