# Supplementary material for: Jumbo circular extrachromosomal elements of methane-oxidizing archaea with variably extensive metabolic and defense gene repertoires
Source: Nat Commun. 2026 Jun 23;17:7878. doi: 10.1038/s41467-026-74423-z (PMC13444090; doi:10.1038/s41467-026-74423-z)
Supplement: Supplementary file 3 [file 41467_2026_74423_MOESM5_ESM.pdf]

nature portfolio

Corresponding author(s): Jill Banfield

Last updated by author(s): May 1, 2026

Reporting Summary

Nature Portfolio wishes to improve the reproducibility of the work that we publish. This form provides structure for consistency and transparency in reporting. For further information on Nature Portfolio policies, see our Editorial Policies and the Editorial Policy Checklist.

Statistics

For all statistical analyses, confirm that the following items are present in the figure legend, table legend, main text, or Methods section.

n/a	Confirmed	
☐	☒	The exact sample size (*n*) for each experimental group/condition, given as a discrete number and unit of measurement
☐	☒	A statement on whether measurements were taken from distinct samples or whether the same sample was measured repeatedly
☐	☒	The statistical test(s) used AND whether they are one- or two-sided<br>*Only common tests should be described solely by name; describe more complex techniques in the Methods section.*
☒	☐	A description of all covariates tested
☒	☐	A description of any assumptions or corrections, such as tests of normality and adjustment for multiple comparisons
☐	☒	A full description of the statistical parameters including central tendency (e.g. means) or other basic estimates (e.g. regression coefficient) AND variation (e.g. standard deviation) or associated estimates of uncertainty (e.g. confidence intervals)
☐	☒	For null hypothesis testing, the test statistic (e.g. *F*, *t*, *r*) with confidence intervals, effect sizes, degrees of freedom and *P* value noted<br>*Give P values as exact values whenever suitable.*
☒	☐	For Bayesian analysis, information on the choice of priors and Markov chain Monte Carlo settings
☒	☐	For hierarchical and complex designs, identification of the appropriate level for tests and full reporting of outcomes
☐	☒	Estimates of effect sizes (e.g. Cohen's *d*, Pearson's *r*), indicating how they were calculated

Our web collection on statistics for biologists contains articles on many of the points above.

Software and code

Policy information about availability of computer code

Data collection	No software was used.
Data analysis	BBTools (https://archive.jgi.doe.gov/data-and-tools/software-tools/bbtools/), hifiasm-meta (v0.13-r308), metaMDBG (v0.3), Prodigal (v2.6.3), MMseqs2 (6f45232ac8daca14e354ae320a4359056ec524c2), GTDB-Tk (v2.3.0), Minimap2 (v2.28-r1209), SAMtools (v1.17), Geneious Prime (2025.0.3), USEARCH (v10), BLASTp (v2.16.0+), HMMER3 (v3.3), eggNOG-mapper, TAfinder 2.0, PSORT (v3.0), LocalColabFold (v1.5.5), Foldseek (version 427df8a6b5d0ef78bee0f98cd3e6faaca18f172d), AlphaFold3, DiGAlign, ComplexHeatmap, CoverM v0.6.1, MODIFI v0.0.1, MAFFT (v7.453), trimAl (v1.4.rev15), IQ-TREE (v2.3.6), iTOL, ChimeraX (1.7.1), featureCounts (v2.0.6), MUMmer (v4.0.0rc1), shinyCircos-V2.0.

For manuscripts utilizing custom algorithms or software that are central to the research but not yet described in published literature, software must be made available to editors and reviewers. We strongly encourage code deposition in a community repository (e.g. GitHub). See the Nature Portfolio guidelines for submitting code & software for further information.

Data

Policy information about availability of data

All manuscripts must include a data availability statement. This statement should provide the following information, where applicable:

- Accession codes, unique identifiers, or web links for publicly available datasets
- A description of any restrictions on data availability
- For clinical datasets or third party data, please ensure that the statement adheres to our policy

Metagenomic and metatranscriptomic sequencing reads have been uploaded to the NCBI Sequence Read Archive (SRA) with the BioProject accession number:

PRJNA1404836. The genomes of Methanoperedens and associated ECEs can be accessed at Figshare: https://doi.org/10.6084/m9.figshare.32069694. The custom protein structure models generated in this study using ColabFold and AlphaFold3 have been deposited in Figshare under the DOI: https://doi.org/10.6084/m9.figshare.32069694. Previously published structure models used as references are available in public databases: the Protein Data Bank (PDB, https://www.rcsb.org/) and the AlphaFold database (version 2025-07-31; https://alphafold.ebi.ac.uk/). Source data are provided with this paper.

Research involving human participants, their data, or biological material

Policy information about studies with human participants or human data. See also policy information about sex, gender (identity/presentation), and sexual orientation and race, ethnicity and racism.

Reporting on sex and gender	Not relevant
Reporting on race, ethnicity, or other socially relevant groupings	Not relevant
Population characteristics	Not relevant
Recruitment	Not relevant
Ethics oversight	Not relevant

Note that full information on the approval of the study protocol must also be provided in the manuscript.

Field-specific reporting

Please select the one below that is the best fit for your research. If you are not sure, read the appropriate sections before making your selection.

☐ Life sciences ☐ Behavioural & social sciences ☒ Ecological, evolutionary & environmental sciences

For a reference copy of the document with all sections, see nature.com/documents/nr-reporting-summary-flat.pdf

Ecological, evolutionary & environmental sciences study design

All studies must disclose on these points even when the disclosure is negative.

Study description	Explore jumbo, archaeal extrachromosomal genetic elements in wetland soil and rice field.
Research sample	Wetland soil in Lake County, California and rice field soil in Butte County, California, USA.
Sampling strategy	L-D.S., B.K., L.E.V.A., and J.F.B. collected the soil samples using soil probe according to the manual. This was a routine sampling once a year. The sample size was determined based on our prior sampling experience at this site.
Data collection	L-D.S., B.K., L.E.V.A., and J.F.B. recorded the data in a lab notebook.
Timing and spatial scale	From 2021 to 2022. Depths of 80 - 110 cm below the surface at a wetland site and 20 - 30 cm at a rice field.
Data exclusions	No data were excluded.
Reproducibility	Samples were collected once a year. The extrachromosomal elements and microorganisms in this study were constantly detected in those samples.
Randomization	Samples were not allocated into groups.
Blinding	Not relevant.

Did the study involve field work? ☐ Yes ☒ No

Reporting for specific materials, systems and methods

We require information from authors about some types of materials, experimental systems and methods used in many studies. Here, indicate whether each material, system or method listed is relevant to your study. If you are not sure if a list item applies to your research, read the appropriate section before selecting a response.

Materials & experimental systems

n/a	Involved in the study
x	Antibodies
x	Eukaryotic cell lines
x	Palaeontology and archaeology
x	Animals and other organisms
x	Clinical data
x	Dual use research of concern
x	Plants

Methods

n/a	Involved in the study
x	ChIP-seq
x	Flow cytometry
x	MRI-based neuroimaging

Plants

Seed stocks

Not relevant

Novel plant genotypes

Not relevant

Authentication

Not relevant
