# Supplementary material for: Jumbo circular extrachromosomal elements of methane-oxidizing archaea with variably extensive metabolic and defense gene repertoires
Source: Nat Commun. 2026 Jun 23;17:7878. doi: 10.1038/s41467-026-74423-z (PMC13444090; doi:10.1038/s41467-026-74423-z)
Supplement: Supplementary file 4 [file 41467_2026_74423_MOESM6_ESM.pdf]

nature portfolio

Peer Review File

Jumbo circular extrachromosomal elements of methane-oxidizing archaea with variably extensive metabolic and defense gene repertoires

Corresponding Author: Professor Jillian Banfield

This file contains all reviewer reports in order by version, followed by all author rebuttals in order by version.

Version 0:

Reviewer comments:

Reviewer #1

(Remarks to the Author)
This manuscript reports the discovery of seven circular jumbo extrachromosomal elements (ECEs) and one integrative ECE associated with Methanoperedens archaea, representing a significant contribution to our understanding of archaeal genetic diversity. The genomic and metabolic analyses are comprehensive; however, several methodological and experimental limitations need to be addressed to strengthen the conclusions.

Major Concerns:
1. Methodological Description and Reproducibility
While the discovery of these ECEs constitutes the central claim of the paper, the methodological workflow for identifying and validating these elements is insufficiently described. Critical steps—including how circular ECEs were distinguished from chromosomal fragments or assembly artifacts, the specific criteria for validating the 409-kbp integrative element, and the integration of HiFi/short-read data—are scattered throughout the text rather than presented as a systematic pipeline.

Please provide a dedicated Methods section or supplementary protocol detailing the complete workflow from raw reads, assembly, binning, to ECE identification, including specific parameters and filtering thresholds. Specify how potential repeat-induced circularization artifacts were excluded during assembly validation. A flowchart illustrating this pipeline is essential for clarity. Additionally, the code of ECE identification should be publicly available to ensure reproducibility.

2. Lack of Experimental Validation
The majority of conclusions regarding metabolic complementation (e.g., nitrite reductase, sulfur reductase, and methane oxidation genes) rely solely on computational predictions and sequence similarity. Without functional assays or genetic experiments, claims that these ECEs "expand host metabolic capacity" remain speculative. While acknowledging the difficulty of cultivating Methanoperedens, the authors should either (a) provide experimental evidence (e.g., enzyme assays, heterologous expression) for key metabolic genes, or (b) significantly tone down claims regarding functional complementation, framing them as hypotheses requiring future validation.

3. Limited Transcriptomic Evidence
The metatranscriptomic analysis covers only two ECE samples, with one showing an order-of-magnitude lower expression than the other. This limited sampling precludes robust conclusions about ECE activity across different environmental conditions or growth states.

Overall, the discovery of these jumbo ECEs is valuable for the field, but the manuscript requires substantial revision to improve methodological transparency and to appropriately qualify claims lacking experimental support.

Reviewer #2

(Remarks to the Author)
In their article entitled “Jumbo circular extrachromosomal elements of methane-oxidizing archaea with variably extensive

metabolic and defense gene repertoires", Shi and colleagues characterized six circular extrachromosomal elements (ECEs) >= 200 kb (named jumbo ECEs by the authors) associated with Methanoperedens, and one similar element integrated in a Methanoperedens genome. This genus corresponds to anaerobic methanotrophic (ANME) archaea. In addition, seven plasmids < 200 kb also related to Methanoperedens, and 11 Methanoperedens complete genomes (including 10 newly sequenced), were also characterized in the study.

Little is known about large ECEs in archaea, and this study represents a very significant insight, especially since ANME play a major role in biogeochemical cycles. Jumbo ECEs add to the diversity of large ECEs in Methanoperedens, alongside Borgs, large linear ECEs recently described by the same group (2022), from which they differ in several key features, including their genome topology. I suggest accepting the manuscript with minor modifications.

Notably, the jumbo ECEs represent a novel type of ECE in archaea, which the authors position in the continuum between plasmids and minichromosomes, as these elements have host-like replication machinery and encode many metabolic genes and defense systems that could expand their hosts' functional repertoire and increase their fitness. The authors suggest that jumbo ECEs could have evolved through persistent incorporation of host DNA.

The article is clearly written, and the analyses are robust, very rich and well-conducted. They rely mostly on metagenome sequencing, genome reconstruction, comparative genomics, phylogeny and structure prediction, as well as metatranscriptomics. They clearly show the association between jumbo ECE and Methanoperedens. At least 14 distinct genes or gene families/operons related to metabolisms and encoded in at least one jumbo ECE are described, along with 6 genes or gene families/operons related to defense systems (including CRISPR-Cas systems and an extracellular contractile injection system). The expression of jumbo ECE genes is shown for two ECEs using metatranscriptomic data from sediment samples, and expressed genes include some related to DNA replication, to the nitrogen cycle or to CO metabolism.

I have only minor suggestions, listed below.

In the introduction, it could be useful to briefly mention the environmental significance of ANME and/or Methanoperedens, to better contextualize the study for readers less familiar with this group.
Regarding the bioinformatic methods, it would be useful to provide more detailed information on the workflows and parameters in the supplementary materials (e.g. for metagenomic assembly, metatranscriptomic analysis, etc). The scripts could be made public if possible (e.g. on GitHub), to further support reproducibility.

A summary table of the main genes/operons analyzed (metabolism, defense systems, replication, etc) showing their presence and absence across the diverse ECEs and their hosts could be useful. It could be provided in the supplementary materials.

For many figures, the text is very small. It would likely be too small to read when printed at standard size (e.g. Figure 2, Figure 3 and several supplementary Figures, such as Supplementary Fig. 10 etc).

Lines 110-114: "Based on the DNA modification patterns and sequence similarity, Methanoperedens_40-02_00 and very closely related strains apparently host five of the ECEs, two of which have > 200 kbp genomes (Supplementary Figs. 3 and 4). Three of the ECEs are likely hosted by strains of Methanoperedens_43-46_00, and a Mp_4-11MB_42_23 strain is the likely host for one jumbo ECE." It would be useful to explicitly list the ECEs, in each case (although this can be inferred from the figures).

Lines 131-133: "DNA replication origin recognition genes (Orc1) were identified in all jumbo ECEs (including the integrated one) and in some of the smaller ECEs (Supplementary Fig. 4)." Supplementary Fig. 4 is probably not the right one, as it shows the DNA methylation patterns. Maybe Table S4 Supplementary Fig. 6?

Lines 195-197: "Jumbo ECEs encode genes related to nitrogen compound transformations. A copper-containing nitrite reductase that is never found in the host Methanoperedens is encoded by 233kb_41_10 and 283kb_39_10 (Supplementary Fig. 11)" In the Supplementary Fig. 11, a protein related to Candidatus Methanoperedens can be seen in close vicinity to the ECEs (HEY9204852.1_Candidatus_Methanoperedens_sp.). Could the authors comment on that? Does this protein have a predicted function and is the taxonomic assignation reliable?

Lines 357-358: "This distinguishes the ECEs from chromids, which utilize plasmid replication and partitioning systems". It could be useful to also explicitly state whether jumbo ECEs can be minichromosomes or not and why. In the same line, in the abstract, it is stated (lines 31-33): "Overall, our results reveal a spectrum of jumbo ECEs of Methanoperedens, ranging from plasmid-like to minichromosome-like." And at the end of the discussion (lines 418-420): "we conclude that they fall on a spectrum between minichromosomes and plasmids, revealing a continuum in the evolution of different ECE types associated with Methanoperedens archaea." The authors could explicitly classify each jumbo ECE within this continuum, whenever possible, specifying the criteria on which this classification is based.

Line 422 and followings, section on DNA extraction, sequencing, and processing: could you please indicate whether individual or cross-assemblies were performed? (I assume individual…). It could also be useful to provide more information on the samples, if available, in the supplementary materials (physico-chemical parameters for instance).

Line 443: "We initially focused on the unclassified" It could be useful to indicate more precisely what unclassified means.

Does it refer to the type of element, to the lack of host prediction?

In Figure 2: “C-terminal Ala-Val-Ser-Gly-Gly peptides of the Methanoperedens 837 SAMP1-like protein are colored in yellow.” The yellow color does not provide a good contrast in this figure. Would it be possible to use another color, for example light green?

Open Access This Peer Review File is licensed under a Creative Commons Attribution 4.0 International License, which permits use, sharing, adaptation, distribution and reproduction in any medium or format, as long as you give appropriate credit to the original author(s) and the source, provide a link to the Creative Commons license, and indicate if changes were made.
In cases where reviewers are anonymous, credit should be given to 'Anonymous Referee' and the source.
The images or other third party material in this Peer Review File are included in the article’s Creative Commons license, unless indicated otherwise in a credit line to the material. If material is not included in the article’s Creative Commons license and your intended use is not permitted by statutory regulation or exceeds the permitted use, you will need to obtain permission directly from the copyright holder.
To view a copy of this license, visit https://creativecommons.org/licenses/by/4.0/

RESPONSE TO REVIEWERS' COMMENTS

Reviewer #1 (Remarks to the Author):

This manuscript reports the discovery of seven circular jumbo extrachromosomal elements (ECEs) and one integrative ECE associated with Methanoperedens archaea, representing a significant contribution to our understanding of archaeal genetic diversity. The genomic and metabolic analyses are comprehensive; however, several methodological and experimental limitations need to be addressed to strengthen the conclusions.

Response: We thank the reviewer for appreciating the significance of our manuscript. We have now addressed all the comments, particularly added a detailed methodological description and toned down the claims regarding the ECE function. In the point-by-point responses below, each comment is *italicized*, followed by our response in normal text. Quotations of the changes are shown in blue here and in the revised manuscript text.

Major Concerns:

1. Methodological Description and Reproducibility

While the discovery of these ECEs constitutes the central claim of the paper, the methodological workflow for identifying and validating these elements is insufficiently described. Critical steps—including how circular ECEs were distinguished from chromosomal fragments or assembly artifacts, the specific criteria for validating the 409-kbp integrative element, and the integration of HiFi/short-read data—are scattered throughout the text rather than presented as a systematic pipeline.

Please provide a dedicated Methods section or supplementary protocol detailing the complete workflow from raw reads, assembly, binning, to ECE identification, including specific parameters and filtering thresholds. Specify how potential repeat-induced circularization artifacts were excluded during assembly validation. A flowchart illustrating this pipeline is essential for clarity. Additionally, the code of ECE identification should be publicly available to ensure reproducibility.

Response: We thank the reviewers for the suggestion. We have now added a flowchart detailing the complete workflow from DNA sequencing to ECE identification, and a comprehensive description of each step in the Methods section "**Identification and curation of archaeal ECE genomes**". Particularly, we explained how to distinguish ECE genomes from chromosomal fragments, how to preclude assembly artifacts (e.g., repeat-induced circularization artifacts), how to curate genomes to completion, and how to

validate the integrative element. In the meantime, we also provided the references to the publicly available scripts in the Supplementary Text.

Lines 433-487 in the revised manuscript:

Identification and curation of archaeal ECE genomes

Extracted DNA was sequenced using the PacBio HiFi long-read technology and the Illumina short-read platform (Supplementary Fig. 1). Low-quality raw reads were trimmed and/or removed using BBDuk (Supplementary Text). Long reads from individual samples were assembled into scaffolds using hifiasm-meta (v0.13-r308)[69] and metaMDBG (v0.3)[70]. Genes in assembled scaffolds were predicted by Prodigal (v2.6.3) using the DNA code 11[71]. Taxonomy was assigned to each protein using mmseqs easy-taxonomy (version: 6f45232ac8daca14e354ae320a4359056ec524c2)[72] based on the best hit in the GTDB v226[73]. Putative self-circular genomes produced by PacBio HiFi assemblies were classified using GTDB-Tk (v2.3.0) against the prokaryotic reference database (v226)[74]. Genomes that could be confidently assigned to taxonomy were considered microbial. We first focused on the GTDB-Tk unclassified, likely non-microbial genomes that were > 200 kbp in length, lacked rRNAs and ribosome components, and had proteins with best matches to those of Archaea, as typical of archaeal jumbo ECEs.

We mapped PacBio HiFi reads to these putatively circular, archaeal jumbo ECE genomes using Minimap2 (v2.28-r1209) with the map-hifi mode and default parameters[75] (Supplementary Text). Generated mapping SAM files were converted to BAM formats by SAMtools (v1.17)[76] and visualized in Geneious Prime (2025.0.3). Extended read portions beyond the genome ends were extracted and remapped to the genome (Supplementary Fig. 2). Exclusive matching of extended read portions at one genome end to the other indicates circularization. Long reads were also used to resolve the correct assembly path of genome regions that contain long repeats that were shorter than the PacBio read lengths.

The real circular jumbo ECE genomes were then mapped by Illumina short reads from the same sample using BBMap (https://github.com/BioInfoTools/BBMap). PacBio-derived genome base calls were inspected at every position and curated when unsupported using both long and short reads. After these steps, the genomes were considered complete. The GC skew and cumulative GC skew patterns were calculated. When the cumulative skew indicated bidirectional replication, the minimum cumulative skew was used to select the origin of replication (in an intergenic region).

The complete jumbo ECE genomes were used to identify related genome fragments, including < 200 kbp sequences. Proteins in the complete genomes were blasted against the local assembly database to search for related scaffolds. Long reads and short reads

were mapped to recruited scaffolds, as described above. The reads placed at scaffold ends and the unplaced paired Illumina reads were used to extend the sequences. In some cases, the extended sequences enabled a join with another scaffold. This process was repeated until the further extension of one end of the genome overlapped with the other end of the genome, indicating circularization[77]. All the genomes were subjected to curation using long and short reads to ensure the accuracy of each base call.

Dramatic changes in read coverage are always interesting. In this case, we observed a huge coverage dip when mapping reads to a *Methanoperedens* genome. To capture reads that partially matched the reference genome in the region of coverage drop, we remapped reads allowing 30% mismatches per read. Read segments that showed inconsistency with the genome at one side perfectly matched the other side, indicating that an element was integrated in a subset of the *Methanoperedens* population (Supplementary Fig. 3). The read inconsistency also defined the boundary of the integrative element. Reads that matched the reference sequence without these discrepancies represent the *Methanoperedens* genome with the integrative element.

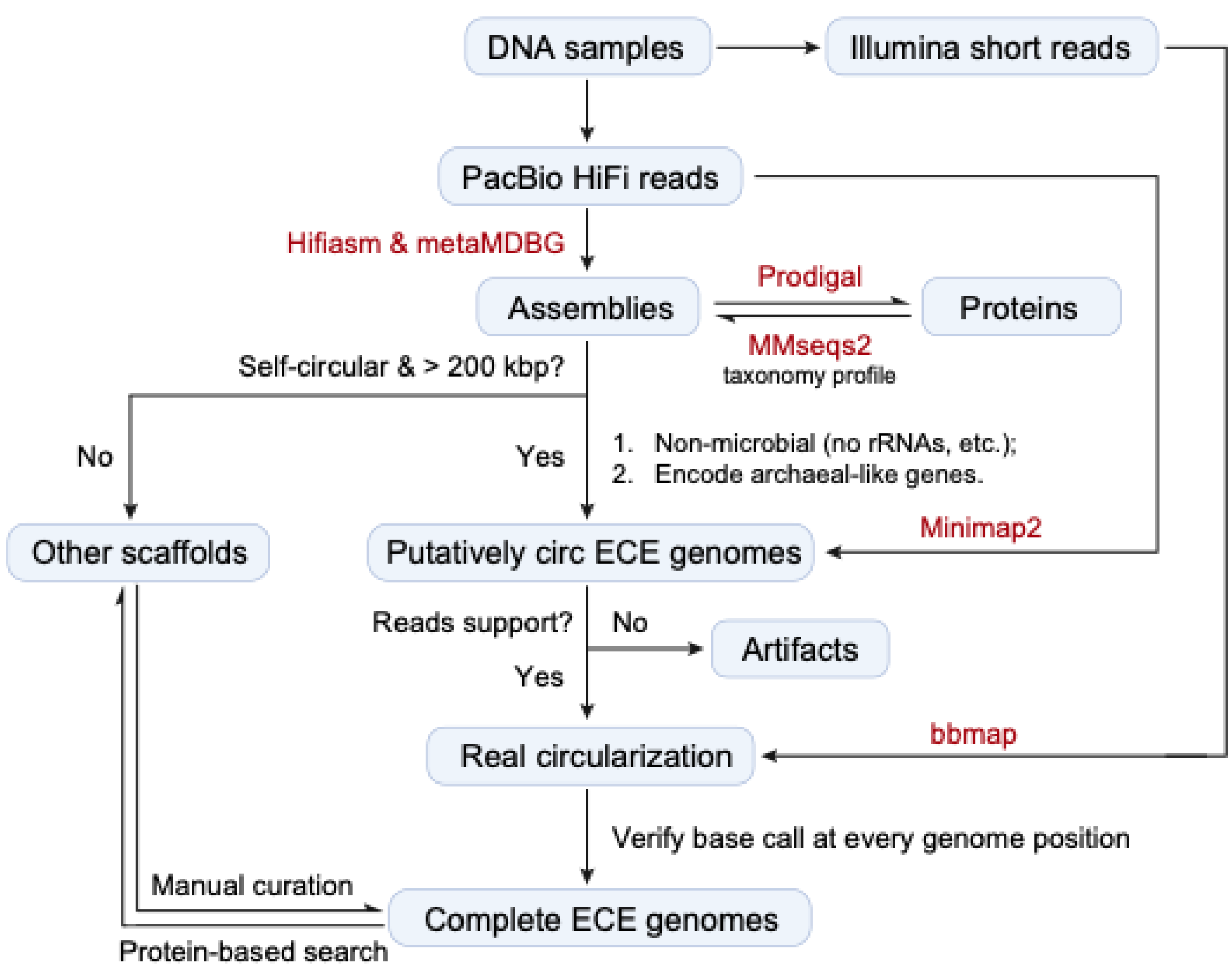

Supplementary Figure 1. Workflow for identification of archaeal ECE genomes. Names in red indicate tools used in the protocol. Detailed information can be found in the **Methods**.

2. Lack of Experimental Validation

The majority of conclusions regarding metabolic complementation (e.g., nitrite reductase, sulfur reductase, and methane oxidation genes) rely solely on computational predictions and sequence similarity. Without functional assays or genetic experiments, claims that these ECEs "expand host metabolic capacity" remain speculative. While acknowledging the difficulty of cultivating Methanoperedens, the authors should either (a) provide experimental evidence (e.g., enzyme assays, heterologous expression) for key metabolic genes, or (b) significantly tone down claims regarding functional complementation, framing them as hypotheses requiring future validation.

Response: We have toned down the claims regarding the ECE function. For example, we inferred that "Jumbo ECEs **may** expand *Methanoperedens* metabolic capacity", "the jumbo ECE sequence variants likely differ in their redox potentials, thus **could** expand the host's ability to pass electrons to extracellular mineral electron acceptors", and "the gene variants **may** expand metabolic capacity due to different substrate specificities and/or reaction rates".

3. Limited Transcriptomic Evidence

The metatranscriptomic analysis covers only two ECE samples, with one showing an order-of-magnitude lower expression than the other. This limited sampling precludes robust conclusions about ECE activity across different environmental conditions or growth states.

Response: We did not conclude anything about ECE activity across different environmental conditions or as a function of host growth states. Instead, we stated that "the newly identified ECEs encode, **and in some cases express**, metabolic genes such as tetrahydromethanopterin S-methyltransferase exclusively involved in methane metabolism, and genes for nitrogen and sulfur compound transformations". Thus, we have not made changes to the text in response to this comment.

Overall, the discovery of these jumbo ECEs is valuable for the field, but the manuscript requires substantial revision to improve methodological transparency and to appropriately qualify claims lacking experimental support.

Response: Thank you very much for your constructive comments.

Reviewer #2 (Remarks to the Author):

In their article entitled "Jumbo circular extrachromosomal elements of methane-oxidizing archaea with variably extensive metabolic and defense gene repertoires", Shi and colleagues characterized six circular extrachromosomal elements (ECEs) >= 200 kb (named jumbo ECEs by the authors) associated with Methanoperedens, and one similar element integrated in a Methanoperedens genome. This genus corresponds to anaerobic methanotrophic (ANME) archaea. In addition, seven plasmids < 200 kb also related to Methanoperedens, and 11 Methanoperedens complete genomes (including 10 newly sequenced), were also characterized in the study.

Little is known about large ECEs in archaea, and this study represents a very significant insight, especially since ANME play a major role in biogeochemical cycles. Jumbo ECEs add to the diversity of large ECEs in Methanoperedens, alongside Borgs, large linear ECEs recently described by the same group (2022), from which they differ in several key features, including their genome topology. I suggest accepting the manuscript with minor modifications.

Notably, the jumbo ECEs represent a novel type of ECE in archaea, which the authors position in the continuum between plasmids and minichromosomes, as these elements have host-like replication machinery and encode many metabolic genes and defense systems that could expand their hosts' functional repertoire and increase their fitness. The authors suggest that jumbo ECEs could have evolved through persistent incorporation of host DNA.

The article is clearly written, and the analyses are robust, very rich and well-conducted. They rely mostly on metagenome sequencing, genome reconstruction, comparative genomics, phylogeny and structure prediction, as well as metatranscriptomics. They clearly show the association between jumbo ECE and Methanoperedens. At least 14 distinct genes or gene families/operons related to metabolisms and encoded in at least one jumbo ECE are described, along with 6 genes or gene families/operons related to defense systems (including CRISPR-Cas systems and an extracellular contractile injection system). The expression of jumbo ECE genes is shown for two ECEs using metatranscriptomic data from sediment samples, and expressed genes include some related to DNA replication, to the nitrogen cycle or to CO metabolism.

Response: We thank the reviewer for the very positive comments.

I have only minor suggestions, listed below.

In the introduction, it could be useful to briefly mention the environmental significance of ANME and/or Methanoperedens, to better contextualize the study for readers less familiar with this group.

Response: Thanks for the suggestion. We have added a brief introduction of *Methanoperedens*, which reads (lines 46-52 in the revised manuscript):

Jumbo archaeal ECEs include circular minichromosomes of Haloarchaea (sometimes designated megaplasmids[20–23]) and one circular, undescribed ~285 kbp putative plasmid of *Methanomethylovorans hollandica* (CP003363). Recently, linear giant Borgs with genome lengths up to 1.1 Mb were reported and predicted to replicate in uncultured *Methanoperedens*[24], a group of archaea that can directly couple anaerobic oxidation of methane to the reduction of nitrate, iron, manganese, and arsenate, to mitigate methane emissions[25-29].

Regarding the bioinformatic methods, it would be useful to provide more detailed information on the workflows and parameters in the supplementary materials (e.g. for metagenomic assembly, metatranscriptomic analysis, etc). The scripts could be made public if possible (e.g. on GitHub), to further support reproducibility.

Response: We thank the reviewer for the suggestion. We have added a comprehensive, detailed description of the bioinformatic methods in the Methods section. We also provided the references to the publicly available scripts in the Supplementary Text. Please see the response to Reviewer #1.

A summary table of the main genes/operons analyzed (metabolism, defense systems, replication, etc) showing their presence and absence across the diverse ECEs and their hosts could be useful. It could be provided in the supplementary materials.

Response: We have now added a new supplementary table (Supplementary Data 6) summarizing the presence and absence of the main genes/operons across the ECEs and the host *Methanoperedens*.

For many figures, the text is very small. It would likely be too small to read when printed at standard size (e.g. Figure 2, Figure 3 and several supplementary Figures, such as Supplementary Fig. 10 etc).

Response: We have increased the font size in Figures 2 and 3, and Supplementary Figures 4, 11 (originally Supplementary Fig. 10), 12, 16, and 18. The important information in all the figures should now be readable at standard printed size.

Lines 110-114: "Based on the DNA modification patterns and sequence similarity, Methanoperedens_40-02_00 and very closely related strains apparently host five of the ECEs, two of which have > 200 kbp genomes (Supplementary Figs. 3 and 4). Three of the ECEs are likely hosted by strains of Methanoperedens_43-46_00, and a Mp_4-

11MB_42_23 strain is the likely host for one jumbo ECE." It would be useful to explicitly list the ECEs, in each case (although this can be inferred from the figures).

Response: We have rephrased the sentence, which reads now (lines 111-117 in the revised manuscript):

Based on the DNA modification patterns and sequence similarity, Methanoperedens_40-02_00 and very closely related strains apparently host five of the ECEs (340kb_39_43, 272kb_39_43, 87kb_39_4, 79kb_38_7, and 65kb_40_4), two of which have > 200 kbp genomes (Supplementary Figs. 4 and 5). Three of the ECEs (157kb_40_9, 62kb_40_11, and 53kb_39_7) are likely hosted by strains of Methanoperedens_43-46_00, and a Mp_4-11MB_42_23 strain is the likely host for one jumbo ECE (269kb_42_21).

Lines 131-133: "DNA replication origin recognition genes (Orc1) were identified in all jumbo ECEs (including the integrated one) and in some of the smaller ECEs (Supplementary Fig. 4)." Supplementary Fig. 4 is probably not the right one, as it shows the DNA methylation patterns. Maybe Table S4 Supplementary Fig. 6?

Response: Sorry for the mistake. It has been corrected now. Thank you for your careful reading!

Lines 195-197: "Jumbo ECEs encode genes related to nitrogen compound transformations. A copper-containing nitrite reductase that is never found in the host Methanoperedens is encoded by 233kb_41_10 and 283kb_39_10 (Supplementary Fig. 11)" In the Supplementary Fig. 11, a protein related to Candidatus Methanoperedens can be seen in close vicinity to the ECEs (HEY9204852.1_Candidatus_Methanoperedens_sp.). Could the authors comment on that? Does this protein have a predicted function and is the taxonomic assignation reliable?

Response: Sharp eyes! The reference protein mentioned above was not characterized in the literature. We blasted the protein sequence against the NCBI nr database and our local assembly database, and found no homologs from other *Methanoperedens*. However, other proteins in the same scaffold do have homologs in other *Methanoperedens* genomes. Thus, it could be a *Methanoperedens* scaffold that encodes a putative copper-containing nitrite reductase. We have rephrased the sentence in the text accordingly. Thank you for pointing this out.

Lines 198-201 in the revised manuscript:

A copper-containing nitrite reductase that is rarely found in the host *Methanoperedens* is encoded by 233kb_41_10 and 283kb_39_10 (Supplementary Fig. 12).

Lines 357-358: "This distinguishes the ECEs from chromids, which utilize plasmid replication and partitioning systems". It could be useful to also explicitly state whether jumbo ECEs can be minichromosomes or not and why. In the same line, in the abstract, it is stated (lines 31-33): "Overall, our results reveal a spectrum of jumbo ECEs of Methanoperedens, ranging from plasmid-like to minichromosome-like." And at the end of the discussion (lines 418-420): "we conclude that they fall on a spectrum between minichromosomes and plasmids, revealing a continuum in the evolution of different ECE types associated with Methanoperedens archaea." The authors could explicitly classify each jumbo ECE within this continuum, whenever possible, specifying the criteria on which this classification is based.

Response: We agree that it would be ideal if we could explicitly classify each jumbo ECE, as the reviewer suggested. However, the definition of minichromosomes remains a debate. What we can confidently conclude is that these jumbo ECEs are neither canonical plasmids nor chromids, given that they utilize host-like replication machinery and encode numerous metabolic genes. Consequently, whether or not they can be categorized as minichromosomes is an open question. Thus, we prefer to describe them as elements falling on a spectrum between minichromosomes and plasmids.

Line 422 and followings, section on DNA extraction, sequencing, and processing: could you please indicate whether individual or cross-assemblies were performed? (I assume individual…). It could also be useful to provide more information on the samples, if available, in the supplementary materials (physico-chemical parameters for instance).

Response: Yes, individual assemblies were performed. Some element concentrations of the samples from the same site were described in our previous paper, so we did not report these data in this manuscript. We have added a citation of that paper to the site description in the Methods.

Lines 427-429 in the revised manuscript:

Soil samples were collected at depths ranging from 80 to 110 cm below the surface at a wetland site in Lake County, California, USA. Geochemical profiles of this site were described previously[24].

Lines 437-439 in the revised manuscript:

Long reads from individual samples were assembled into scaffolds using hifiasm-meta (v0.13-r308)[69] and metaMDBG (v0.3)[70].

Line 443: "We initially focused on the unclassified" It could be useful to indicate more precisely what unclassified means. Does it refer to the type of element, to the lack of host prediction?

Response: We used GTDB-Tk to classify Pacbio self-circular genomes. Some genomes were classified as "Unclassified", suggesting that they were likely not microorganisms and might be ECEs. We have rephrased the sentence.

Lines 443-449 in the revised manuscript:

Putative self-circular genomes produced by PacBio HiFi assemblies were classified using GTDB-Tk (v2.3.0) against the prokaryotic reference database (v226)[74]. Genomes that could be confidently assigned to taxonomy were considered microbial. We first focused on the GTDB-Tk unclassified, likely non-microbial genomes that were > 200 kbp in length, lacked rRNAs and ribosome components, and had proteins with best matches to those of Archaea, as typical of archaeal jumbo ECEs.

In Figure 2: "C-terminal Ala-Val-Ser-Gly-Gly peptides of the Methanoperedens 837 SAMP1-like protein are colored in yellow." The yellow color does not provide a good contrast in this figure. Would it be possible to use another color, for example light green?

Response: We have changed the yellow color to light green and attached the updated figure below, for your convenience. Thank you very much for all your constructive comments.

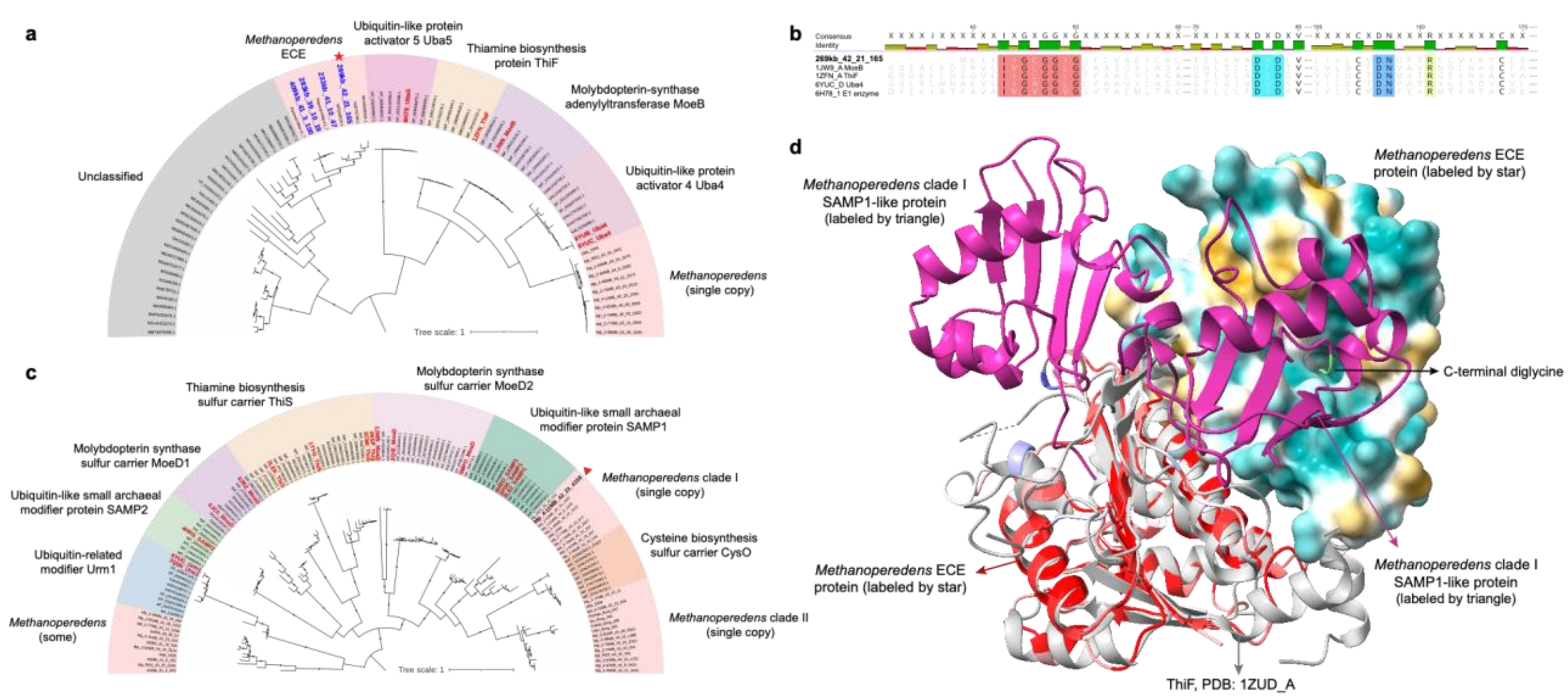

Figure 2. Sequence and structural analyses of ThiF and ThiS family proteins in Methanoperedens and jumbo ECEs. (**d**) … C-terminal Gly-Gly peptides of the *Methanoperedens* SAMP1-like protein are colored in light green.
